# Vascular Burden, dementia biomarkers and Neuropsychiatric Symptoms in Dementia

**DOI:** 10.1002/alz.088048

**Published:** 2025-01-03

**Authors:** Cheng‐Sheng Chen, Hsiu‐Fen Lin

**Affiliations:** ^1^ Kaohsiung Medical University Hospital, Kaohsiung Taiwan; ^2^ Kaohsiung Medical University, Kaoshiung Taiwan

## Abstract

**Background:**

Neuropsychiatric symptoms (NPSs) significantly impact outcomes for individuals with dementia, with multiple factors contributing, including neurobiology. This study aims to enhance our understanding of the neurobiology of NPSs by examining the links between vascular burdens, dementia biomarkers, and NPSs.

**Method:**

Patients with dementia receiving outpatient services were included. The Neuropsychiatric Inventory (NPI) assessed NPSs. Vascular burdens were evaluated through clinical vascular diseases and white matter hyperintensities (WMH) severity using the Fazekas score in brain MRI. Additionally, ApoE genotypes and visual ratings of medial temporal atrophy (MTA) in brain MRI were used as dementia biomarkers.

**Result:**

Forty‐five dementia patients participated, with the majority having Alzheimer’s disease (80%), followed by vascular dementia (8.9%), dementia with Lewy bodies (8.9%), and frontotemporal dementia (2.2%). The results indicated associations between depression symptoms and vascular diseases (p = 0.03), as well as anxiety symptoms (p = 0.03) and the depression/apathy cluster (p = 0.03) with MRI Fazekas scores. Regarding dementia markers, agitation/aggression was associated with ApoE ε4 (p = 0.02).

**Conclusion:**

The findings suggest specific associations between vascular burden and ApoE ε4 with certain NPSs, linking vascular burden to depression and anxiety, and agitation to ApoE ε4.

